# How personality factors, coping with identity-stress, and parental rearing styles contribute to the expression of somatic complaints in emerging adults in seven countries

**DOI:** 10.3389/fpsyt.2024.1257403

**Published:** 2024-05-15

**Authors:** Inge Seiffge-Krenke, Heribert Sattel

**Affiliations:** ^1^ Department of Psychology, University of Mainz, Mainz, Germany; ^2^ Department of Psychosomatic Medicine and Psychotherapy, Technical University Munich, University Hospital rechts der Isar, Munich, -Germany

**Keywords:** somatic complaints, personality, coping style, identity stress, parental rearing style

## Abstract

**Objective:**

Somatic complaints are frequently named by emerging adults in many countries, but psychological factors contributing to the high level of these often medically unexplained symptoms have received little attention. This study examines the influence of shared risk factors on somatic complaints in a culturally diverse sample.

**Methods and measures:**

In a cross-cultural survey study of 2,113 emerging adults (mean age = 22.0 yrs.; *SD* = 2.04) from seven countries (France, Germany, Turkey, Greece, Peru, Pakistan, and Poland) personality variables, parental rearing styles, coping abilities as well as identity-related stress were assessed. In a second step we successively entered these variables in hierarchical linear mixed models, controlling for country and gender effects and their respective interaction, in order to determine their impact on the level of somatic complaints across countries.

**Results:**

All these dimensions varied extensively between all countries, with females reporting higher levels of somatic complaints than men in several countries. Despite this variation, our findings demonstrate a general and stable influence of neuroticism, openness, parental rearing styles, coping abilities and identity-related stress on somatic complaints across countries.

**Conclusion:**

Findings support the use of a general intervention model that includes appropriate coping strategies for emotion regulation, but also encourages support seeking for age-specific problems in dealing with identity stress during the transition to adulthood. In addition, this intervention model should be adjusted for a specific culture and gender.

## Introduction

A growing number of emerging adults from different countries have difficulties during the transition into the adult world (1). The postponement of relevant developmental tasks and the oscillation between transitory and inconsistent states with respect to work, partnership, and residential status (2–4) have a negative impact on health. While several studies substantiated an association between transitional stress and increased psychopathology (5–7), the possible effects on the emergence of body symptoms are largely unclear.

Somatic complaints are body-related sensations or perceptions with subjectively experienced unpleasant qualities. Their number and quality can be understood as an indicator for general bodily distress (8). Fatigue, headaches, backache, stomachache, and nervousness/agitation constitute frequently occurring complaints (9), with sleep problems being particularly frequent in emerging adults (10). Only in about 3-10% of these somatic complaints, a medical cause could be found (11). Overall, the prevalence of somatic complaints among college students ranges from 15 to 60% (11). Studies in different countries revealed quite high rates of somatic complaints in non-clinical samples of emerging adults, covering the ages of 18 to 30, for example in the German-wide representative survey among university students (12), the Transitions from Education to Employment (TREE) study in Switzerland (9), the Iceland representative cohort of 15-year-olds follow-up till 23 years (13), the large internet-based survey in Sweden (14), and the National Longitudinal Survey of Children and Youth, which followed Canadian children from birth to early adulthood (15). Further studies in China (16), Japan, the US (17), and Israel (18) confirmed gender and ethnic differences in somatic complaints among university students.

So far only 4% of the studies on emerging adult samples have examined populations outside of Europe and North America (19), and they did not investigate somatic complaints. Other studies that examined influencing factors on somatic complaints lumped together emerging adults with other age groups (18 to 65 years) and did not analyze age-specific risk factors for this particular age group (20–22). To explain so-called ‘medically unexplained symptoms’, a complex etiological model can be used, including biological, social and psychological factors (23). To better understand the age-specific factors that are associated with the hidden expression of bodily distress via somatic complaints in emerging adults, we utilize an integrated approach focusing on social and psychological factors. Hence, we examined the role that personality, identity, coping, and social resources play in the emergence of somatic complaints amongst emerging adults in seven countries.

A major developmental task that has shifted from adolescence to young adulthood is the reconceptualization of identity (1), which causes a lot of stress. Associations between delayed identity development and identity stress were found among young people in many Western countries, with high rates in internalizing disorders such as depression, (e.g., 24, 25). Among emerging adult patients, the rate of identity stress was nearly double, compared to non-conspicuous age-mates (26). It is yet unknown how identity stressors are related to the quite high rate of somatic complaints seen in emerging adults in different countries.

The individuals’ agency and cultural aspects also may play a role in how identity challenges are dealt with (27). Identity stressors (such as a perceived difficulty in obtaining the desired employment, insecurity about career choice, and the compatibility of family start-up and professional career) have increased during the last decade (28, 29). Thus, investigating the efforts that emerging adults in different countries undertake to cope with identity-related stressors is important. Adaptive coping styles (e.g., actively tackling the issues at stake, reflection, and seeking support) are a prerequisite for successful progress across emerging adulthood (30).

Normative development in early adulthood also reflects growth in the direction of greater maturity in personality (31). During this period, individuals across the world tend to become more agreeable, more conscientious, and less neurotic (32). Based on the Big Five model of personality, both longitudinal and cross-sectional studies have shown a marked increase in emerging adults’ openness to new ideas and experiences. Given the tendency to postpone markers of adulthood such as establishing firm partnerships (4) or a professional career (33) into the third decade, is an increase in somatic complaints related to the non-normative timing of various transitions and what is the role of personality in this context?

In many countries, young people continue to reside with their parents (34). Research in Western industrialized nations substantiate that dysfunctional parental rearing styles can impair autonomy development and lead to an increase in psychopathology. More specifically, a tendency of parents to provide (too much) protection and support has come into focus (35). Such hovering parental behavior or “helicopter parenting” has been found in samples in central Europe and in North America (36, 37). It could be demonstrated that other parenting styles such as exhibiting psychological control or monitoring the adult children (38, 39), are associated with higher rates of psychopathology in Western societies. Research is still lacking on whether this applies to parents in other countries (40). Furthermore, the impact of these parenting styles on somatic complaints of the offspring is unclear.

## Current study and hypotheses

Analyzing factors that can explain high levels of somatic complaints is important, as afflicted emerging adults will seek help in the health system. Moreover, from a cultural psychopathology perspective (41), sensitivity to cultural differences in somatic complaints is gaining importance, given the increasing number of foreign patients in inpatient and outpatient settings in Western countries (42). This study focuses on somatic complaints in emerging adults from different countries. We have taken a broad approach by including social and psychological variables that are particularly typical of this phase of development and that are presumed to have an impact on body complaints. Given the importance of identity formation for emerging adults’ functioning, the investigation of stress in the area of identity seems particularly important to investigate. In the present study we also focus on the coping strategies that individuals use in dealing with identity- related stressors and challenges. Further, certain parenting styles, which are empirically closely linked to problems in identity development, such as anxious rearing or psychological control, might predispose individuals in different cultural contexts to exhibit body complaints. We assume that identity stress and, for example, psychological control of parents tend to be risk factors for the development of body complaints, while an active coping style and supportive parental behavior may be protective factors that help buffer the effect of identity stress. Since previous studies have also used personality factors to explain high rates of body complaints, we will also integrate these variables into our approach, although we assume that their predictive power is rather low. Taken together, our study analyzes how personality factors, identity-related stress, coping with identity stress, and parental rearing styles varies between participants, and, further, based on the potential variation, which factors generalize across countries as potential risk factors for the emergence of somatic complaints.

The present study is part of an ongoing project, in which adolescents and young adults in seven countries were examined with regard to risk factors that lead to impaired psychological and physical health. Based on high scores in psychopathology and somatic complaints, adolescence is regarded as a window of vulnerability (43, 44). Likewise, due to high rates in certain psychopathological disorders and in physical ailments, emerging adulthood is regarded as second window of vulnerability (6, 45). A first study on 2415 adolescents (46) focused on somatic complaints. We found that certain key aspects of parenting were related to the occurrence of somatic complaints across countries. In another study on 2113 emerging adults (47), the focus was on psychopathology, more specifically on internalizing and externalizing symptoms, and the high variation in these symptoms across countries. In this study, we partialled out identity- and parenting-related covariates to gain a more genuine pattern of country and gender-specific effects. Pre-existing country differences for psychopathology disappeared when risk factors such as identity stress or dysfunctional maternal parenting behavior were controlled for. Building on that, an important aim of the current study was to detect, across countries, shared risk factors which may help explain the high rates of somatic complaints in emerging adults from different countries. For reasons of comparability, instruments from previous studies were used. Overall, this approach may help to identify similarities and differences in risk factors for somatic as compared to psychological symptoms. A better understanding of those risk factors can be considered as important prerequisite for planning prevention and intervention.

We approach our aim by investigating emerging adults from seven countries throughout the world (e.g., France, Germany, Turkey, Greece, Peru, Pakistan, and Poland) with a focus on an age range of 20 to 24 years. All participants were students and lived in large university cities to ensure a roughly comparable developmental context. We included two developing countries (Pakistan and Peru), two countries with recent political and economic changes (Turkey and Greece), and three countries from Europe (France, Germany, Poland), where economic situation, future perspectives and parenting practices potentially may vary despite their regional vicinity (see, for more details, 47).

A first research question was to analyze culture- and gender-specific differences in somatic complaints. In addition, we explored differences in personality variables, identity stress, coping with identity stress, and parental rearing styles (support, psychological control, and anxious monitoring) across the seven cultural samples. Although there is research suggesting some cross-cultural universality of personality traits (48), we expect also culture-specific differences in the personality variables (49, 50). For example, some cultures are more outgoing and sociable, and these differences may buffer the effects of stress during the transitional phase, while cultures with a less open, more strict approach and fixed norms potentially augmented the already existing stress of the transitional phase. In the context of different future opportunities and levels of freedom to decide, we expect that potential identity stress will be expressed in the form of somatic complaints, especially in cultures where open expression of distress due to strict society and family rules is less accepted, for example in Pakistan. Further, we expect that difficult parenting practices such as intrusive and controlling parental behavior may contribute to the rate of somatic complaints seen in non-clinical emerging adults especially from Western countries like France and Germany.

Second, in search for universal factors that generalize across countries, we developed hierarchical linear mixed models to estimate the impact of these respective variables on somatic complaints. As there is research suggesting strong gender differences in many but not all countries, with females reporting higher levels in somatic complaints as compared to males, we intend to analyze and control for those possible effects and their interaction. This can be achieved by the application of linear mixed models with country as nested grouping factor. We expected that high identity-related stress would significantly be associated with somatic complaints across cultures, whereas an adaptive coping style such as support seeking when dealing with identity stress serve as protective factor across cultures. Possibly, some personality variables such as openness and extraversion and, further, supportive relationships with friends and parents may serve as protective factors in the overall model. We did expect that parental psychological control and anxious monitoring, which have been found to be risk factors for psychological health in emerging adults from Western countries (see 35, 47, 51), would be associated with higher somatic complaints in emerging adults from France, and Germany, but we did not expect that such a potential negative effect generalizes across cultures.

## Method

### Participants

We assessed sample sizes of *N* = 300 per country and ensured that the emerging adults came from a similar educational and developmental context. Data were collected from a sample of 2,113 emerging adults from seven countries (France, Germany, Turkey, Greece, Peru, Pakistan, and Poland). **Table 1** provides an overview of the sample’s demographics by country. Mean age and age variance were reasonably well balanced among the samples (M=22.0; SD=2.04). The gender ratio was also well balanced for all countries but Turkey. There were marked differences in family structure and size between countries. Most emerging adults lived in two-parent families. The number of children per family varied widely across countries, with the lowest in Germany and the highest in Pakistan. Further, friendship status, self-rated socioeconomic status and parental education differed significantly between countries.

**Table 1:** Sociodemographic sample characteristics for the different countries (means, standard deviations or frequencies).

		Total N=2048	France	Germany	Greece	Pakistan	Peru	Poland	Turkey	Sig. p=^1^
			230	351	296	293	302	295	281	-
	*Range*		M (SD)	M (SD)	M (SD)	M (SD)	M (SD)	M (SD)	M (SD)	
**Somatic complaints**	**(0-16)**	5.15 (3.4)	7.69 (3.9)	6.44 (1.6)	3.79 (2.9)	4.27 (3.1)	5.13 (3.5)	4.72 (3.8)	4.31 (3.5)	**.001**
**Somatic complaints (men)**		4.39 (3.00)	6.86 (3.4)	6.25 (1.8)	2.81 (2.4)	4.23 (2.6)	4.58 (3.5)	3.43 (3.1)	3.27 (2.2)	**.007 ^2^**
**Somatic complaints (women)**		5.51 (3.5)***	7.78 (4.0)	6.56 (1.4)	4.35 (3.00)***	4.29 (3.3)	5.63 (3.5)**	5.41 (3.9)***	4.47 (3.6)**	
**Age**		21.8 (2.4)	20.9 (1.7)	23.9 (2.6)	22.2 (3.2)	21.1 (1.2)	21.2 (1.5)	20.8 (1.2)	21.9 (2.7)	**.001**
**No of siblings**		1.78 (1.5)	1.74 (1.8)	1.11 (0.9)	1.42 (1.2)	3.6 (1.7)	1.43 (1.0)	1.45 (1.2)	2.00 (1.4)	**.001**
**Gender female (percent)**		1394 (68.1%)	209 (90.9%)	216 (61.5%)	189 (63.9%)	184 (62.8%)	160 (53.0%)	192 (65.1%)	244 (86.8%)	**.001**
**Status of family of origin^3^**										**.001**
Two-parent		82.0%	86.5%	90.3%	78.4%	88.5%	80.1%	80.5%	91.2%	
Single parent		11.1%	13.0%	8.8%	17.9%	9.7%	17.2%	14.0%	7.6%	
**Self-rated SES**										**.001**
High		20.8%	^4^	7.4%	12.4%	32.1%	18.2%	28.2%	29.9%	
Average		74.0%	^4^	84.3%	82.1%	65.5%	77.5%	63.9%	68.3%	
Low		5.1%	^4^	8.3%	5.5%	1.7%	4.3%	7.9%	1.8%	
**Parental education**										**0.001**
Primary/secondary		9.1%	15.7%	9.7%	5.1%	4.8%	1.0%	15.1%	19.7%	
High-school		30.1%	30.9%	17.7%	40.2%	16.3%	13.7%	39.2%	30.5%	
Undergraduate/graduate		60.1%	53.5%	72.6%	54.7%	76.5%	85.0%	44.2%	49.5%	
**Close same-sex friend**										**0.001**
No		13.9%	31.7%	6.3%	^4^	18.4%	10.7%	17.7%	3.9%	
Yes		86.1%	68.3%	93.7%	^4^	81.6%	89.3%	82.3%	96.1%	

^1^p-values printed in bold indicate statistically significant values (p<0.05).

^2^Anova, controlling for age and gender x country interaction.

^3^Numbers for small remaining categories not reported here.

^4^Data not reported.

### Instruments

#### Somatic complaints

The participants’ level of somatic complaints was assessed with the respective scale from the Young Adult Self Report (YASR; 52). We used in this study the scale of somatic complaints, which comprises the following 7 items: stomach aches, tiredness, feeling tense, feeling physically weak, headaches, rashes/skin problems, nausea, and dizziness, to be answered following a ternary answer format (1 = *not true*, 2 = *somewhat or sometimes true*, 3 = *often or very often true*). The YASR has been used in several countries with good reliability (see 53). In the current study, Cronbach’s alpha for somatic complaints was .89 across countries.

#### Identity-related stress

Emerging adults’ stress was measured with the Problem Questionnaire (PQ; 54), where we used the nine items pertaining to the domain of identity-related stress (e.g., a strong motivation to discover one’s needs, perceived difficulty in obtaining desired employment, insecurity about career choice and family and work-life balance). A sample item is, “I found it difficult to discover what profession really suits me.” The participants rated the identity-related stressors on a 5-point scale (1 = *not stressful at all* to 5 = *highly stressful*). The PQ has been used frequently across countries with good reliability (47). In the current study, Cronbach’s alpha amounted to .91 for identity-related stress across all countries.

#### Coping with identity-related stress

Participants completed the Coping Across Situations Questionnaire (CASQ; 54), which assesses 20 coping strategies across the identity domain on a binary scale (0 = *strategy not used*; 1 = *used*). These strategies can be compiled into three coping styles: Negotiating and Support Seeking, Reflection, and Emotional Control. The first style, termed *Negotiating and Support Seeking*, comprises nine items such as “I discuss the problem with my parents.” or “I try to solve the problem with the help of my friends.” The second style, termed *Reflection*, includes six items such as “I think about the problem and try to find a solution.” Five items measure the third style, termed *Emotional Control*, such as “I withdraw because I cannot change anything in anyway”. The CASQ has been used frequently across countries with good reliability [see Persike et al. (47)]. In the current study, Cronbach’s alpha amounted to .89 for Negotiating and Support seeking .78 for Reflection and .82 for Emotional Control across all countries.

#### Big five personality

The BFI-S (55) assessed the Big Five Personality by means of three items per dimension (Conscientiousness, Extraversion, Agreeableness, Openness, and Neuroticism). In this 15-item instrument the statements were rated on a 7-point Likert scale ranging from 1=*does not apply to me at all* to 7=*applies to me perfectly*. In the current study, Cronbach’s alpha amounted to .88 for Conscientiousness, .77 for Extraversion, .86 for Agreeableness, .79 for Openness, and.81 for Neuroticism across all countries.

#### Perceived parental behavior

Participants completed an instrument that comprised 17 items, assessing a variety of aspects of perceived parental behavior, which are rated on a 5-point scale (1 = *not applicable* to 5 = *very appropriate*). The items were separately answered for mothers’ and fathers’ rearing styles. Five items stemmed from the Adolescent Family Process measure (AFP; 56), such as “My mother/father often supports me”. Furthermore, six items from Barber (38) were used to record parental psychological control (“My mother/father no longer talks to me when I disagree with her/him.”), as well as six items from a measure designed by Kins and colleagues (39) to measure parental overprotection, in particular anxious monitoring (“My mother/father monitors each of my steps, if I want to be alone.”). Reliability amounted to α= .89 for parental support, α= .81 for parental psychological control, and α= .83 for anxious rearing in the current sample.

### Procedure and data acquisition

In all countries, the assessments were conducted on students in university cities to limit variance due to differences in education and urbanization. The responsible ethics boards in each country approved the study. Participants did not receive any incentives to participate. Participants obtained consent forms several days prior to data collection, 90% gave their written consent to participate in the study. About 13% of emerging adults were absent on the day of assessment, resulting in an overall dropout rate of about 23%. All assessments were conducted in a group setting.

In order to ensure cross-cultural validity and equivalence, regular meetings with the collaborators, senior and junior researchers from all seven countries took place, in which the items of the relevant measures were translated into the official language in each country and then back translated into English. Measurement invariance (MI) analyses are available for all instruments used in the study (see 46, 47).

### Data analyses

We determined between-country differences for sociodemographic and psychological variables using an analysis of variance for continuous variables and chi-square tests for ordinal or nominal data. The influence of gender differences between countries for our primary outcome somatic complaints was analysed by introducing gender, country, a country x gender interaction term, and age as independent variables and somatic complaints as the dependent variable. Then, we analysed gender differences for each country separately with independent sample t-tests.

In order to determine the influence of sociodemographic and psychological variables on somatic complaints across all countries, consecutive linear mixed models were estimated (57). These models allow to mix fixed with random factors, with the latter usually representing nested factors as – in our study – countries. In a first step, country, gender, country x gender interaction, presence of a close same-sex friend and age were entered (block 1). Successively we added personality dimensions (block 2), followed by parental rearing styles (block 3), coping styles (block 4) and, finally, identity-related stress (block 5). Data were checked for multicollinearity, and variance inflation factors varied between 1.00 and 1.69, indicating no reasonable multicollinearity (58). All independent variables with the exception of country and country x gender interaction were assumed to exert fixed effects. Whether a set of variables improved the fit of the model was proven by a statistically significant change of the Akaike information criterion (AIC). The level of significance was set to the conventional α = .05 (two-tailed). All data analyses were carried out with SPSS 26.0.

## Results

### Variation between countries in the study variables

The psychological sample characteristics by country can be seen in **Table 2**. The average level of somatic complaints varied significantly between emerging adults from the participating countries. The mean values ranged from 3.79 in Greece to more as double (7.69) in France. Additionally, an overall influence of gender could be observed, with higher scores in somatic complaints in females. Overall, the magnitude of this difference was specific for the countries, with no significant gender differences for France, Germany and Pakistan and continuously increasing and statistically significant differences for Peru, Turkey, Greece and Poland.

**Table 2:** Psychological sample characteristics for the different countries (means, standard deviations or frequencies).

		Total	France	Germany	Greece	Pakistan	Peru	Poland	Turkey	Sig. p ^1^
**Personality BFI**	Range 1-7	M (SD)	M (SD)	M (SD)	M (SD)	M (SD)	M (SD)	M (SD)	M (SD)	
Conscientiousness		3.28 (0.86)	2.65 (0.88)	2.63 (0.75)	3.84 (0.68)	3.25 (0.71)	3.53 (0.63)	3.37 (0.66)	3.78 (0.82)	**0.001**
Extraversion		3.17 (0.94)	2.97 (1.04)	2.27 (0.65)	3.37 (0.81)	3.19 (0.79)	3.28 (0.88)	3.48 (0.68)	3.85 (0.87)	**0.001**
Agreeableness		3.09 (1.00)	2.17 (0.77)	1.91 (0.44)	3.81 (0.74)	3.44 (0.75)	3.39 (0.69)	3.17 (0.70)	3.85 (0.73)	**0.001**
Openness		3.48 (0.92)	2.54 (0.92)	2.94 (0.47)	3.62 (0.79)	3.70 (1.08)	3.96 (0.7)	3.63 (0.71)	3.92 (0.79)	**0.001**
Neuroticism		3.09 (0.95)	2.24 (0.98)	2.89 (0.7)	3.39 (0.91)	3.06 (1.04)	3.39 (0.8)	3.35 (0.86)	3.17 (0.88)	**0.001**
**Parental rearing styles**	Range 1-5									
Support		3.67 (0.77)	2.69 (0.46)	3.51 (0.33)	3.74 (0.79)	3.9 (0.75)	4.03 (0.74)	3.79 (0.83)	3.85 (0.65)	**0.001**
Psychological. control		2.62 (0.71)	2.62 (0.45)	2.92 (0.49)	2.20 (0.68)	2.98 (0.73)	2.41 (0.71)	2.52 (0.69)	2.64 (0.82)	**0.001**
Anxious monitoring		2.83 (0.71)	2.65 (0.59)	2.64 (0.33)	2.73 (0.70)	3.39 (0.72)	2.77 (0.67)	2.72 (0.79)	2.88 (0.79)	**0.001**
**Coping CASQ**	Range 0-1									
Negotiating and support seeking		0.59 (0.22)	0.57 (0.19)	0.52 (0.18)	0.62 (0.18)	0.54 (0.19)	0.6 (0.32)	0.65 (0.20)	0.68 (0.17)	**0.001**
Reflection		0.62 (0.22)	0.71 (0.18)	0.48 (0.20)	0.64 (0.18)	0.66 (0.18)	0.63 (0.30)	0.61 (0.17)	0.67 (0.19)	**0.001**
Emotional control		0.41 (0.24)	0.32 (0.22)	0.51 (0.22)	0.39 (0.21)	0.35 (0.22)	0.42 (0.37)	0.42 (0.18)	0.45 (0.19)	**0.001**
**Identity related stress (PQ)**	Range 1-5									
		2.48 (0.78)	3.14 (0.71)	2.76 (0.44)	2.38 (0.72)	2.16 (0.82)	2.35 (0.79)	2.20 (0.78)	2.45 (0.78)	**0.001**

^1^anova, effect of country. P-values printed in bold indicate statistically significant values (p<0.05).

Measures for personality characteristics varied widely between emerging adults from the seven countries, with lower values for France and Germany, and values consistently near and above the center of the respective scale for all other countries. Parental rearing styles (support, psychological control and anxious monitoring) varied with small or moderate effect sizes between all countries except support, which was markedly smaller in France as compared to the total mean of the sample. All three measured coping scales (negotiating and support seeking, reflection, emotional control) were distributed similarly for each country around the total sample mean. Identity-related stress revealed to be most prominent in France, whilst all other countries remained in a span indicating small to moderate effect sizes for the respective means, compared to the whole sample.

### Linear mixed models explaining somatic complaints across countries

The hierarchical linear mixed models revealed a statistically significant effect of gender, and tendentially for having a close same-sex friend in the first step, whilst age and country and gender x country interaction, intended as control dimensions, appeared to exert non-negligible effects which, however, did not reach statistical significance (see **Table 3**). The introduction of personality variables resulted in a significant improvement of the observed model fit: Low conscientiousness and high neuroticism were statistically significant associated with more pronounced somatic complaints, with a similar but non-significant effect for openness. When parental rearing styles were included in the model (step 3), a particularly marked increase of the model fit could be observed. All new predictors revealed to be highly influential for the prediction of somatic complaints, with low parental support, and high psychological control and anxious rearing, respectively, being associated with more somatic complaints. A similar effect could be observed in step 4, when coping styles were included into the model. Little use of negotiating and support seeking and inversely, a high use of reflection and emotional control as coping style led to more pronounced somatic complaints. Finally, the inclusion of identity-related stress revealed an additional, statistically significant effect on somatic complaints, again underlined by an improvement of the model. All associations identified in the previous steps of the hierarchical model remained stable, with the exception of conscientiousness, which fell short to reach significance and was replaced by openness in the final model.

**Table 3:** Hierarchical multilevel models on predicting somatic complaints.

Steps/blocks and variables	Step 1	Step 2	Step 3	Step 4	Step 5	B (95% CI)
	(F; p-value)	(F; p-value)	(F; p-value)	(F; p-value)	(F; p-value)	(fixed effects)
AIC	9026.41	8772.67***	1657.00***	1629.382***	1586.146***	
1st step/block: sociodemographics						
Male gender	**9.02 (0.027)**	**9.20 (0.027)**	**10.2 (0.02)**	**7.99 (0.033)**	**8.69 (0.029)**	-0.12 (-0.23;-0.02)
Age (years)	0.42 (0.519)	0.10 (0.756)	0.01 (0.954)	0.01 (0.947)	0.01 (0.951)	0.00 (-0.01;0.01)
Close same-sex friend	3.50 (0.061)	1.70 (0.193)	1.30 (0.25)	0.79 (0.375)	0.56 (0.453)	0.02 (-0.03;0.07)
Country^1^	1.44 (0.149)	1.34 (0.181)	1.31 (0.189)	1.25 (0.211)	1.14 (0.240)	–
Gender x country^1^	1.08 (0.279)	1.05 (0.294)	1.17 (0.242)	1.22 (0.223)	1.19 (0.251)	–
2nd step/block: personality						
Openness		3.61 (0.058)	3.38 (0.066)	**4.07 (0.044)**	**3.98 (0.046)**	-0.02 (-0.05;-0.001)
Conscientiousness		**8.56 (0.003)**	**4.15 (0.042)**	**4.32 (0.038)**	1.36 (0.244)	-0.02 (-0.04;0.01)
Extraversion		1.99 (0.159)	1.31 (0.253)	1.38 (0.240)	0.03 (0.873)	0.00 (-0.02;0.02)
Agreeableness		1.37 (0.243)	1.00 (0.317)	0.61 (0.434)	0.67 (0.413)	-0.01 (-0.04;0.02)
Neuroticism		**31.1 (0.001)**	**19.5 (0.001)**	**11.96 (0.001)**	**4.14 (0.042)**	0.02 (0.001;0.04)
3rd step/block: parental rearing styles						
Support			**21.7 (0.001)**	**19.7 (0.001)**	**13.3 (0.001)**	-0.06 (-0.09;-0.03)
Psychological control			**7.47 (0.006)**	**7.10 (0.008)**	**4.82 (0.028)**	0.04 (0.004;0.07)
Anxious rearing			**35.0 (0.001)**	**35.6 (0.001)**	**29.3 (0.001)**	0.09 (0.06;0.13)
4th step/block						
Negotiating and Support Seeking				**18.46 (0.001)**	**15.4 (0.001)**	-0.20 (-0.30;-0.10)
Reflection				**10.05 (0.002)**	**8.01 (0.005)**	0.14 (0.04;0.24)
Emotional control				**11.32 (0.001)**	**7.57 (0.006)**	0.12 (0.03;0.20)
5th step/block						
Identity-related stress					**39.78 (0.001)**	0.09 (0.06;0.12)

AIC, Akaike information criterion, lower values indicate better model fit.

***improvement of model fit for successive models (based on the AIC) p < 0.001. F- and p-values printed in bold indicate statistically significant values (p<0.05).

^1^For random effects Wald z was determined.

## Discussion

The aim of our present study was – in a first step – to demonstrate that culture- and gender-specific differences between Western and Non-Western countries exist in multiple domains: in somatic complaints as our primary outcome, as well as in personality characteristics, identity stress and coping, and parental rearing styles across the seven cultural samples. In a second step we could demonstrate that the associated factors personality, stress, coping and parental rearing style exerted an overarching effect on bodily distress, in that way that male gender, openness and low neuroticism, a high level of parental support and lower levels of parental psychological control and anxious rearing, a certain pattern of coping styles and low levels of identity-related stress were independently associated with less somatic complaints, when initially controlled for country and country x gender interaction.

Many Western countries have large proportions of immigrant emerging adults as well as young people coming for a certain period of time either to study or for an apprenticeship. Sensitivity to cultural differences in somatic complaints is gaining importance (41), taking into account the increasing number of foreign patients in inpatient and outpatient settings (42), which were frequently diagnosed with high rates in somatic complaints and in somatization (59). Given the cultural diversity, it is important to analyze shared risk factors that can explain high levels of somatic complaints, and can serve to design basic interventions that generalize across cultures. In this study personality variables, parental-rearing behavior, coping styles and identity stress were selected as potential risk factors in order to determine their general contribution to the level of somatic complaints in emerging adults from seven countries (France, Germany, Greece, Pakistan, Peru, Poland, and Turkey).

The question of which of the variables examined have a universal impact on somatic complaints was examined by hierarchical linear mixed models across countries, controlling for country and gender effects as well as for their respective interaction. Our findings demonstrate a general and stable influence of all these predictors on somatic complaints, when successively entered in hierarchical multilevel models. Overall, each set of predictors contributes to the overall predictive ability and relative importance on somatic complaints. Although parents’ supportive behavior was perceived quite differently in emerging adults from the seven countries, high rates of parental support were uniformly related to low levels of somatic complaints. Evidently, parental support served as a protective factor not only for internalizing and externalizing psychopathology (47, 60). In several studies, besides a lack of parental support, high rates of parental psychological control were associated with higher levels of depression and anxiety (61, 62). Our results extend on these findings and demonstrate that the negative effect of psychological control and, additionally, of anxious parental monitoring, on emerging adults’ health is not limited to internalizing and externalizing psychopathology (47), but generalize to somatic complaints. Further, this negative impact, which was found in samples in central Europe or North America (38, 63), also apply to emerging adults from North Europe, South America, and Asia. Such hovering parental behavior seems to limit the exploration behavior of emerging adults, hinders the development of competence in dealing with age-specific tasks (64), and can therefore be experienced as stressful with corresponding effects on physical health.

Of further interest in the present study were key aspects of personality development in this age group and their impact on somatic complaints. With respect to conscientiousness, similar to Lam &McBride-Chang (30), our findings were inconclusive; the desire to do a task well, and to take obligations to others seriously was only statistically significant till step 4, but not in the final model. However, two personality variables were consistently associated - at least tendencially significant – with somatic complaints across countries: Openness (which was linked with less somatic complaints) and neuroticism (which was linked with more somatic complaints). These findings are in accordance with studies and a meta-analysis that neuroticism had a negative effect on well-being (65, 66), specifying the important role in increasing physical ailments. Of note is the positive impact of openness, suggesting that the openness to explorations in the areas of work and relationships, which is developmentally appropriate in the transitional phase, is associated with fewer somatic complaints.

In this context it should be noted that a meta-analysis summarizing research in European, North- and South American countries substantiated that identity development was postponed into emerging adulthood (67) and has changed in quality with high exploration and low commitment in work, relationships, and values. Based on Erikson’s (68) approach, numerous studies in the following decades demonstrated that delayed identity development is related to several mental disorders, such as anxiety and depression (see 7 for an overview). In fact, in our study, identity stress was one of the strongest predictors of somatic complaints across countries. This suggests that rapid social and technological changes, an increasing plurality of values and growing economic insecurity have made the development of young people’s identity more insecure and stressful in many countries (47, 69, 70), with a strong impact not only on psychopathology, but also on physical health.

Empirical evidence also suggests that identity stressors during the transition to adulthood may overwhelm already compromised coping capacities (45, 71). Our study indicates that negotiating and seeking support as a coping style were associated with lower levels of somatic complaints. In contrast, emotional control and reflection served as a risk factor with high levels in both coping styles contributing to high levels of somatic complaints. Reflection is generally an adaptive style of coping (64), but too much reflection resulting in ruminative exploration (61) seems to be unfavorable. Our findings are in line with other studies (72, 73), which demonstrate a beneficial effect of open expression of emotions, when suffering from somatic complaints. Overall, the current study support ideas of an increasing similarity between participants from different countries being in the same developmental phase (74); they seem to share similar risk factors that can affect physical health. The vanishing of cultural effects (47) and of culture-bound syndromes (75) must be considered, not only in terms of psychopathology, but also in terms of somatic complaints.

As in earlier studies of our project (46, 47), we found variation in most variables between emerging adults from the seven countries. In spite of different political, social, and family backgrounds, and in spite of varying stress levels, the coping style of emerging adults from all seven countries was quite uniformly characterized by high levels in negotiating and support seeking as well as reflection about possible solutions. There was more divergence with respect to emotional control, suggesting that culture-specific standards with regard to an open outlet of emotions prevail (72, 76). We also found culture-specific variation in parenting. Overall, in most countries besides France, parental support was the most frequent parental style. In accordance with other studies, we noticed quite high levels of perceived parental support reported by emerging adults from Peru and Turkey (40, 77). Pakistani emerging adults reported quite high scores in psychological control, confirming findings of a very tight bonding in this country (78). As reported in Persike et al. (47), gender effects in some of these variables were measured.

Together, our results show that potentially bothering somatic symptoms can be found in normative samples of emerging adults in many countries of the world and that persons concerned, despite all diversity, share certain risk factors. Knowledge regarding the influence of a specific culture may additionally help practitioners and researchers in the context of public health care.

### Limitations and future directions

Overall, this study makes several unique contributions to our understanding of emerging adults’ somatic complaints in different countries, but it has also several limitations. First, the sample consisted of university students and therefore might not generalize to non-student populations, to immigrant and to poor youth in the diverse countries. However, the homogenous sample (students from large university cities) was necessary for detecting country specific differences. Thus, our results are not generalizable to emerging adults who are in the job market or unemployed. Therefore, future studies should also include emerging adults from the same countries with a higher variation in SES, education, professional choices, and living circumstances. It can be assumed that young people who suffer from economic crisis, who are unemployed or have precarious working conditions will exhibit higher rates in body complains. One can further assume that their identity stress level is higher and the parental support systems potentially impaired. In addition, other variables such as the developmental status regarding identity may play a role which needs to be considered in future research. Next, a notable limitation is the cross-sectional nature of the study which precludes causal inferences, since it did not allow to assess change over time or potential bidirectional influences. Future research may consider uncovering the complex interplay between personality variables, coping with identity stress and parental rearing styles over time and how it shapes an emerging adult’s health both across and between countries. Even though we carried out a cross-cultural study to take a closer look at varying cultural influences, we have focused in our present analysis on common, culture-overarching influencing factors. In fact, our findings underline the existence of shared risk factors on somatic complaints when examined over all countries. However, the linear mixed models may vary greatly from country to country, which should be analyzed in future studies. Another limitation was that data were obtained via emerging adults’ self-reports, which may have been influenced by perceived norms in each country. Follow-up research would benefit from using several respondents and a multi-method approach (including interviews and observations) in order to achieve a deeper understanding of how culture affects the links between somatic complaints and the respective variables. Nevertheless, future studies should examine the relationship between different disorders of psychopathology and somatic complaints, which can occur mutually as well as distinctly (79). Finally, findings of this study may not be generalizable to somatic complaints in mid- and later life because participants of this study are relatively young (22 years old, on average) and in a specific developmental phase with unique characteristics (29).

## Conclusion

Somatic complaints are crucial clinical warning signs, which, if not tended to, bear some risk to persist into later adulthood, and herald subsequent somatization (59). Further, untreated somatic symptoms may result in frequent consultation of medical services without clear medical treatment regimen, encompassing a high risk for diverse unsuccessful or even harmful medical encounters, and may predispose or reinforce higher levels of functional somatic disorders (23) or psychopathology in the long run (80). Somatic complaints are the product of complex interactions, involving multiple biological and psychosocial factors. In research and clinical use, social and psychological factors have becoming increasingly important, adding to our knowledge about bodily and brain processes. The inclusion of personality variables, parental rearing styles, coping abilities, and identity-related stress as factors in this study reflects a comprehensive approach to understanding the cause of somatic complaints in several cultural contexts.

Our findings have implications for clinical interventions, as they add to our understanding of the etiological mechanism underlying body complaints and demonstrate universals that have been found for emerging adults in several countries. Identity formation has become a central developmental task in emerging adults, with failing to master this task leading to health problems such as depression and anxiety (7). In this study, identity stress and maladaptive coping with identity stress turned out as important risk factors for somatic complaints that were shared by emerging adults in many countries. Parental support, openness for new experiences and an open expression of emotion had a buffering effect on somatic complaints. Specifically, our results suggest that interventions designed to target coping processes with identity stress as well as seeking support from others might be helpful as a basic intervention approach. It was surprising to what extent coping competence and a supportive, nonintrusive parenting style was linked with low levels of body complaints in students from Western and non-Western countries likewise. Promoting the ability to seek help when problems arise and to appropriately reflect on problem solutions without ruminating emerged as key universals in emerging adults across countries. These universal targets should be complemented by culture-specific interventions suitable for the respective country of origin. Such culture-specific interventions could relate to dealing with specific negative emotions and leaning to seek and accept help. Taken together, our research points to similarities in risk factors for somatic complaints, an important prerequisite for planning prevention and intervention on a culturally sensitive base.

## Data availability statement

The original contributions presented in the study are included in the article/supplementary materials, further inquiries can be directed to the corresponding author.

## Ethics statement

Ethical approval was not required for the study involving humans in accordance with the local legislation and institutional requirements. Written informed consent to participate in this study was obtained from the participants or the participants’ legal guardians/next of kin in accordance with the national legislation and the institutional requirements/s.

## Author contributions

IS: Conceptualization, Data curation, Funding acquisition, Investigation, Methodology, Project administration, Resources, Validation, Writing – original draft, Writing – review & editing. HS: Formal analysis, Methodology, Writing – original draft, Writing – review & editing.
